# Cyberbullying perpetration and victimisation amongst adolescent psychiatric patients at Lentegeur Hospital, South Africa

**DOI:** 10.4102/sajpsychiatry.v28i0.1755

**Published:** 2022-03-31

**Authors:** Mahomed E. Paruk, Rene Nassen

**Affiliations:** 1 Department of Psychiatry, Faculty of Medicine and Health Sciences, Stellenbosch University, Cape Town, South Africa

**Keywords:** cyberbullying, cyber-victimisation, adolescents, psychiatric comorbidity, mental health, South Africa

## Abstract

**Background:**

Cyberbullying is a type of harassment that is perpetrated or experienced by a person or groups of persons via the use of electronic devices, and it frequently occurs amongst young people. Research has shown that cyberbullying is associated with psychiatric comorbidity, which could indicate a need for screening adolescents who present for mental health services.

**Aim:**

This study aimed to determine the prevalence of cyberbullying amongst adolescents. The secondary aim was to determine the Diagnostic and Statistical Manual of Mental Disorders (DSM)-5 psychiatric diagnoses associated with cyberbullying.

**Setting:**

Lentegeur Hospital Child and Adolescent Mental Health Service in the Western Cape, South Africa.

**Methods:**

This cross-sectional study included a convenience sample of 97 participants (sampled from both inpatient and outpatient services) between the ages of 13 years and 18 years. Adolescent assent and parental consent were obtained. Demographic and clinical data were collected from patient folders, and the Revised Cyber Bullying Inventory II was used to assess cyberbullying behaviours.

**Results:**

The overall prevalence rate of some form of cyberbullying in this sample was 56.7%, of which 6.2% were cyberbullies, 20.6% were cyber-victims and 29.9% were cyberbullies and cyber-victims. Female participants were more likely to be involved in cyberbullying than males. The most prevalent primary psychiatric diagnoses in adolescents involved in cyberbullying included major depressive disorder (72.4%), schizophrenia (57.1%) and attention deficit hyperactivity disorder (22%). There was no significant association between cyberbullying and any psychiatric diagnoses.

**Conclusion:**

The high prevalence rate of adolescents involved in cyberbullying suggests that this behaviour is a cause for concern in the South African population. More screening and treatment programmes should be implemented to address this issue.

## Introduction

Cyberbullying is a type of bullying that is perpetrated or experienced by a person or groups of persons via the use of electronic devices. It occurs repetitively^1^ and is intentionally utilised to be aggressive and hurtful to the victim.^2^ There are three categories of involvement in cyberbullying: individuals who are victims, bullies (or perpetrators), and those who are both victims and bullies.^3^ Many victims eventually become bullies.^4^

The prevalence rate of cyber-victimisation amongst adolescents varies from 10% to 40%, with some studies showing a prevalence rate of up to 72%.^4^ Most published literature on cyberbullying emerges from North America and Europe, and there are very little data from Africa. Three recent reports from South Africa show prevalence rates of cyber-victims amongst adolescents to be between 15.2% and 46.7%.^5,6,7^

There is a growing body of literature exploring the negative consequences of being a cyberbully or cyber-victim. Certain research studies have shown that cyberbullies and cyber-victims have worse consequences than traditional bullying.^8,9,10^ Bullying, regardless of the type, has been found to be associated with externalising symptoms, such as aggression, substance use, and having reduced empathy, whilst cyber-victimisation has been found to be associated with internalising symptoms such as depression, anxiety, non-suicidal self-injury, suicidal ideation, suicidal attempts and suicide.^11,12,13^ Cyber-victims are also at an increased risk of developing an adjustment disorder^11^ and a conduct disorder.^12^ Peer relationships become increasingly important amongst adolescents, with peer rejection and problems associated with an array of mental health symptoms.^13^ Cyber-victims may also isolate themselves and feel unhappy, which may lead to behavioural symptoms associated with depression.^14^

A meta-analysis conducted by van Dam that explored traditional bullying and psychosis did not find any association between psychosis and traditional bullying.^15^ There are a very few studies demonstrating the prevalence rates of cyberbullying amongst adolescents with a psychiatric comorbidity. One study showed a prevalence rate of cyberbullying or cyber-victimisation amongst those with intellectual disability to be between 10% and 14%.^16^ Another study on the prevalence of cyber-victimisation amongst participants diagnosed with attention deficit hyperactivity disorder (ADHD) and Asperger’s syndrome cited a prevalence rate of around 21.4% in a study of 33 participants.^17^

A local epidemiological study by Kleintjes et al.^18^ estimated that the prevalence rate of mental disorders amongst children and adolescents in South Africa was 17%. The most common mental disorders include generalised anxiety disorder (GAD, 11%), post-traumatic stress disorder (PTSD, 8%), major depressive disorder (MDD, 8%), schizophrenia (0.5%), bipolar disorder (BD, 1%), ADHD (5%), and conduct disorder (4%).^18^ It is to be observed that this is the only study of its kind conducted in South Africa.

The prevalence estimates of cyberbullying and cyber-victimisation amongst children and adolescents worldwide are limited as there are few studies published. To our knowledge, there also appears to be a lack of information available for the prevalence of cyberbullying and cyber-victimisation amongst mentally ill adolescents, both locally and abroad. However, the high use of technology coupled with high rates of face--to-face bullying across the world suggests a potentially higher prevalence rate than that which is documented. This suggests that surveillance and screening for the presence of cyberbullying may be valuable, considering the known link with adverse emotional outcomes, including suicidal behaviour. Adolescents presenting with mental disorders are a particularly vulnerable population who could potentially be screened for the presence of cyberbullying during a mental health visit and expand the role of the healthcare practitioners to screening, psychoeducation and management of the consequences of being a cyberbully or cyber-victim.^1^

The aim of this study was to establish the prevalence of cyberbullying amongst patients presenting to the child and adolescent psychiatry unit at Lentegeur Hospital, South Africa and to determine whether there were any correlations between specific psychiatric diagnoses and cyberbullying.

## Methods

### Study design

This cross-sectional study used a convenience sample of adolescent patients (aged 13–18 years) who presented to the child and adolescent mental health services at Lentegeur Hospital, Cape Town, South Africa between 01 April 2018 and 30 July 2018.

### Study setting

The child and adolescent mental health service at Lentegeur Hospital is a tertiary-level service that provides ambulatory, an inpatient psychosis recovery, and a therapeutic programme for hospitalised adolescents.

### Study sample

Male and female participants, who were not actively psychotic, suicidal or in a crisis and who were able to provide assent were recruited from both in- and out-patients. OpenEpi software (www.openepi.com) was used to calculate the required sample size using α = 0.05, power = 95% and previous reported data that the incidence of cybervictimisation is about 37% – 47.9%.^19^ With these data we estimated a total sample size of 97 participants. Participants were recruited by the registrar and medical officer working in the child and adolescent unit. During the study period there were a total of 436 outpatient consults and 51 inpatients admissions. A total of 104 participants met the inclusion criteria of the study. Of these, seven declined to participate, resulting in a final sample size of 97 participants.

### Data collection

The demographic and diagnostic data were obtained by applying multiple data collection methods, including an interview by the registrar or medical officer of the parents and the participant and from clinical folders. Demographic information (age, gender, etc.) and psychiatric diagnoses, based on the Diagnostic and Statistical Manual of Mental Disorders (DSM-5)^20^ were obtained from patient files. The primary diagnosis was considered based on the main problem that the patient presented with. For example, many participants had both autism and ADHD, and in all these cases autism was the primary diagnosis. Where this information was missing it was gained from the parents and/or guardian during administration of the survey and recorded. The Revised Cyberbullying Inventory-II (RCBI-II) was used to collect information regarding cyberbullying behaviours, with permission granted by the author. The questionnaire was administered by the primary investigator (M.E.P.) or the medical officer.

### Measures

The RCBI-II measures being either a cyberbully and/or a cyber-victim during the preceding six months.^21^ Participants rated how often they experienced cyberbullying and or cyber-victimisation in relation to ten activities.

Examples of activities include spreading rumours and threatening someone. Participants rated the occurrence of the activities during the previous six months using a four-point Likert scale (1 = never, 2 = once, 3 = twice to three times and 4 = more than three times). Summed scores range from 10 to 40 with higher scores representing higher severity. In this study, participants were categorised as cyberbullies, cyber-victims, cyberbullies and cyber-victims, or neither a cyberbully nor a cyber-victim (uninvolved). To be categorised as a cyberbully and a cyber-victim, participants were required to have reported perpetrating and experiencing at least 1 of the 10 items on the RCBI-II on more than one occasion, as the definition of cyberbullying and cyber-victimisation requires the behaviour to be repeated.^22^ Those in the cyberbully and cyber-victim group were required to meet the criteria for both. Those individuals categorised as uninvolved reported either one behaviour on a single occasion or never been involved in any of the behaviours. This questionnaire has not yet been validated in South Africa. Cronbach’s alpha was performed to check the internal reliability of RCBI test scores, and it was found to be reliable with a score of 0.855. This is comparable with the Cronbach’s alpha value of 0.82, which was found in the original study instrument.^21^

### Data analysis

Continuous variables were summarised as mean and standard deviation (s.d.), whilst nominal variables were summarised as counts and percentages. Pearson’s Chi-square test was used to determine significant relationships between cyberbullying category and nominal variables (gender, in- or out-patient, primary diagnosis, school attendance, parental marital status and primary caregiver). When the results of the Chi-square test were significant, we conducted a post hoc z-test on adjusted residuals with Bonferroni correction. All analyses were performed using Statistical Package for Social Sciences (SPSS), version 25 (IBM Corp., Armonk, NY, United States of America), and statistically significant differences were established at *p* < 0.05.

### Ethical considerations

The study was approved by the Health Research Ethics Committee (HREC) of Stellenbosch University (Study number: S18/01/008) and by the Research and Ethics Committee of the Department of Health (Western Cape Government).

Participation was voluntary, with participants having provided assent and their parents informed consent. All data were anonymised by assigning unique identifiers to each participant. All data were stored in a single Excel file and kept on a password protected computer.

## Results

The demographical and clinical characteristics of adolescents in this study are summarised in Table 1. There were more male (53.6%; *n* = 52) than female participants (46.4%; *n* = 45). The majority of participants attended mainstream schooling (67.0%; *n* = 65) and stayed with at least one parent (72.9%; *n* = 63). Only 9.9% (*n* = 29) had biological parents who were married.

**TABLE 1:** Demographic and clinical characteristics of participants (*N* = 97) showing comparisons between cyberbullying behaviours.

Variables	Total		Cyberbullying categories, *n* (%)								
			Not Involved (*n* = 42, 43.3%)		Cyberbully-cyber-victim (*n* = 29, 29.9%)		Cyberbully only (*n* = 6, 6.2%)		Cyber-victim only (*n* = 20, 20.1%)		*p*
*n*	%	*n*	%	*n*	%	*n*	%	*n*	%		
**Gender**											0.007
Male	52	53.6	31	59.6	11	21.2	2	3.8	8	15.4	
Female	45	46.4	11	24.4	18	40	4	8.9	12	26.7	
**School type**											0. 397
Not in school	17	17.5	8	47.1	6	35.3	1	5.9	2	11.8	
Special school	15	15.5	10	66.7	2	13.3	-	-	3	20.0	
Mainstream schooling	65	67.0	24	36.9	21	32.3	5	7.7	15	23.1	
**Parental marital status**											0.915
Divorced	16	16.5	6	37.5	7	43.8	0	0.0	3	18.8	
Separated	35	36.1	16	45.7	7	20.0	3	8.6	9	25.7	
Co-habiting	1	1.0	-	-	1	100.0	-	-	-	-	
Married	29	29.9	13	44.8	9	31.0	2	6.9	5	17.2	
Widow/widower	16	16.5	7	43.8	5	31.3	1	6.3	3	18.8	
**Primary caregiver**											0.823
Foster care	5	5.2	3	60.0	1	20.0	-	-	1	20.0	
Grandparents	18	18.6	8	44.4	5	27.8	-	-	5	27.8	
Other relative	4	4.1	1	25.0	3	75.0	-	-	-	-	
Father only	7	7.2	4	57.1	2	28.6	1	14.3	-	-	
Mother only	41	42.3	17	41.5	11	26.8	3	7.3	10	24.4	
Both parents	22	22.7	9	40.9	7	31.8	2	9.1	4	18.2	
**Patient type**											0.124
Outpatient	56	57.7	30	53.6	14	25.0	3	5.4	9	16.1	
Inpatient	41	42.3	12	29.3	15	36.6	3	7.3	11	26.8	
**Primary psychiatric diagnosis**											0.122
Major depressive disorder	22	22.7	4	18.2	11	50.0	2	9.1	5	22.7	
Attention deficit hyperactivity disorder	18	18.6	15	88.3	2	11.1	-	-	1	5.6	
Schizophrenia	14	14.4	6	42.9	4	28.6	3	21.4	1	7.1	
Bipolar disorder	6	6.2	1	16.7	2	33.6	-	-	3	50.0	
Autism spectrum disorder	5	5.2	3	60.0	1	20.0	-	-	1	20.0	
Post-traumatic stress disorder	8	8.2	4	50.0	2	25.0	-	-	2	25.0	
**Comorbid psychiatric diagnosis**											0.249
Substance use disorder	12	12.4	3	25.0	5	41.7	1	8.3	3	25.0	
Mild intellectual disability	13	13.4	10	76.9	3	23.1	-	-	1	7.1	
Oppositional defiant disorder	8	8.2	5	62.5	2	25.0	1	12.5	-	-	
Attention deficit hyperactivity disorder	3	3.1	1	-	1	-	-	-	1	-	
Parent–child relational problems	4	4.1	-	-	2	-	1	-	1	-	

The most common primary psychiatric diagnosis was MDD (29.9%; *n* = 29), followed by ADHD (18.6%; *n* = 18), schizophrenia (14.4%; *n* = 14), BD (6.2%; *n* = 6), adjustment disorder (5.2%; *n* = 5) and autism spectrum disorder (ASD, 5.1%; *n* = 5) (Table 1). The most common comorbid psychiatric diagnosis was substance use disorder (SUD, 23%; *n* = 20) (which included alcohol, cannabis, opioid and stimulant use disorders), followed by mild intellectual disability (MID, 17.2%; *n* = 15), and oppositional defiant disorder (ODD, 9.2%; *n* = 8). Other notable comorbid illnesses were ADHD (9.2%; *n* = 8), PTSD (6.9%; *n* = 6) and a DSM 5 v-code of parent–child relational problems (5.7%; *n* = 5). Most participants with ASD (*n* = 4) were not attending mainstream school, 3 had comorbid ADHD, 2 had comorbid GAD, 1 had mild ID and 1 had schizophrenia.

More than half of all participants (56.7%, *n* = 55) were involved in one of the three categories of cyberbullying (Table 1). Almost one-third of the participants (29.9%, *n* = 29) were both cyberbullies and cyber-victims, 20.6% (*n* = 20) were cyber-victims only, and 6.2% (*n* = 6) were cyberbullies only. More female participants (75.6%, *n* = 34) were involved in cyberbullying behaviours than male participants (40.4%, *n* = 21), where 8.9% (*n* = 4) of female participants and 3.8% (*n* = 2) of male participants were cyberbullies. Results from Pearson’s Chi-square tests demonstrated that gender was the only variable significantly associated with bullying type (χ^2^ = 12.24; *p* = 0.007, Table 1). Post hoc analyses with Bonferroni correction found that females were significantly more likely (*p* < 0.001) to be involved in cyberbullying behaviour.

Of those patients who had a primary diagnosis of MDD (*n* = 29), the largest proportion (44.8%) were cyberbullies and cyber-victims. Most patients with ADHD (77.8%, *n* = 14) and ASD (*n* = 3) were not involved in cyberbullying behaviour. The proportions of cyberbully only and cyber-victim only were highest amongst individuals with schizophrenia (*n* = 3) and BD (*n* = 3), respectively. Amongst participants with comorbid PTSD, none were only cyberbullies, *n* = 2 were cyberbullies and cyber-victims, and *n* = 2 were cyber-victims only. The percentage of patients with some form of cyberbullying and with both schizophrenia and a comorbid SUD was 26.8% (*n* = 26).

## Discussion

This is one of the first studies to assess the prevalence of cyberbullying in a sample of adolescent psychiatric patients. It was found that more than half of the participants were involved in cyberbullying behaviour, either as cyber-victims, cyberbullies or cyberbullies and cyber-victims, the majority of which were female participants. This is higher than the global prevalence, which ranges from 10% to 40%.^4^ The reason for the higher prevalence in this study may be because this study’s population included psychiatric patients, whilst the other studies sampled school going children and adolescents presumed to be non-psychiatrically referred.

Almost a third of this study participants were cyberbullies and cyber-victims. This is in keeping with other studies that have found that bullies are often victims first and then progress to perpetrate as retaliation to being victimised. Such bullies would justify their acts as an outlet for their feelings of frustration and harassment.^23^

The prevalence rate of being a cyber-victim only was lower than that reported in previous studies. South African studies by Vodafone and the Department of Justice reported prevalence rates of 24% and 46.8%, respectively.^24,25^ However, direct comparisons between our study and these previous studies may be limited because of differences in study design and chosen definitions of cyberbullying. The differences in prevalence could also be accounted for by different population samples as most of the previous studies were conducted at schools, as opposed to our sample, which comprised adolescents attending psychiatric services at a hospital.

In this study, the prevalence rate of being a cyberbully was 6.2%. It is possible that this could be an underestimation as cyberbullies may minimise the harm they cause.^24^ This may have affected the adolescents’ responses as they may have been embarrassed about disclosing bullying or feared exposure. Previous studies have also shown that adolescents were less likely to admit to being a cyberbully compared with traditional bullying because of a cognitive distortion in which they considered such behaviour as a means of retribution rather than viewing it as a form of perpetration.^25^

The most prevalent psychiatric diagnosis was MDD, which was not unexpected considering the increased expected prevalence of MDD during adolescence.^26^ However, most participants with a diagnosis of MDD had some involvement in cyberbullying, with most being cyberbullies and cyber-victims. Selkie et al.^27^ explored the relationship between cyberbullying behaviours, depression and alcohol use amongst a sample of college attending females. They observed six times increased odds of depression in those who are cyber-victims. The proposed pathophysiology underlying the mechanism whereby being a victim may directly result in depression is poorly understood. It is hypothesised that being a victim of cyberbullying may be considered a stressful life event that may lead to depression.^28,29^ In addition, victims have increased rates of an insecure parental attachment and peer rejection, making them more vulnerable to suffering from MDD.^28^

Only 22.2% of participants with a primary diagnosis of ADHD experienced cyberbullying, which was expected to be higher considering the potential impulse control behaviours (and comorbidities) that youth with ADHD may struggle with, including disruptive and oppositional defiant behaviours.^30^ A study in Finland showed a stronger association between cyberbullying and hyperactivity (17.4% vs. 3.4%); however, the study administered the Strengths and Difficulties Questionnaire only, which is not a clinical diagnostic tool but rather an exploration of emotions and behaviours.^31^ To make a diagnosis of ADHD, the symptoms need to be present in two settings, and information from both parents and teachers are also required.^32^ The previous study may, therefore, not be a true reflection of an increased prevalence of cyberbullying in people with ADHD.

In this study, those with a diagnosis of ODD had a 37.5% prevalence of cyberbullying. As a higher prevalence was once again expected, it was postulated that this lower-than-expected rate of cyberbullying amongst adolescents diagnosed with ODD and ADHD may be because of under-reporting as a result of the lower mean age of participants in the study, the majority of whom were under 15 years.^33^

More than half of participants with schizophrenia had some involvement in cyberbullying. This, too, is in keeping with increased rates of cyberbullying amongst people with psychotic symptoms, as reported by Dooley et al.^12^ However, there appears to be a relative lack of studies exploring the rates of cyberbullying amongst patients diagnosed with schizophrenia. It was postulated that the high prevalence of cyberbullying amongst patients with a diagnosis of schizophrenia in this study may be accounted for by the comorbid SUD. Almost half of participants with a comorbid SUD was involved in cyberbullying. This is in keeping with the problem behaviour theory which hypothesises that adolescents who engage in deviant behaviour such as using illicit substances are more prone to violence.^34^ The study by Gámez-Guadix et al.^35^ also showed an association between cybervictimisation and substance use. Relating this to the study population there is a likelihood of comorbid substance use, however, individual histories of substance use were not extracted from the clinical records.

No statistically significant association between primary psychiatric diagnoses, comorbid diagnoses and cyberbullying behaviours was observed in this study. This may be because of the small sample sizes in individual diagnostic groups. Studies with larger sample sizes and possibly different study design would be required to define these specific relationships more accurately.

Some studies have shown that females are more involved in cyberbullying as it is a type of relational or verbal aggression rather than direct, ‘face-to-face’ bullying, which is more commonly observed amongst boys.^36,37^ This result is in accord with a local study that showed that females were more likely to be victims of bullying than males.^38^ However, there are inconsistencies in the literature related to the prevalence of cyberbullying amongst males and females.^39^ Our sample was collected using convenience sampling and the majority of male participants had a diagnosis of ADHD. Those diagnosed with ADHD in this sample had a lower-than-expected prevalence rate of cyberbullying and this possibly accounted for the overall lower than expected rates of cyberbullying amongst males. On the other hand, it may be likely that males may preferably indulge in face-to-face bullying rather than cyberbullying.^36,37^

## Limitations and recommendations

The findings of this study have limited generalisability as the sample consisted of psychiatrically referred adolescents. This study also relied on self-reporting measures and may be susceptible to recall bias. The use of convenience sampling means that this may not be a true representation of the patient population found at the child and adolescent mental health services unit in Lentegeur Hospital. We suggest a more systematic sampling method. This study was only based on participants’ responses, whereas a study monitoring chat rooms and with direct access to the messages may reveal a more accurate picture. There is also a referral bias as patients with less severe illness may be treated at a district level of care, and thus not be referred to a tertiary unit and would, therefore, not be included in the study. Future studies should include a larger population size in order to better assess the possible association between cyberbullying behaviour and psychiatric diagnoses. The RCBI II has not been validated in South Africa. Given the high prevalence of cyberbullying in our sample, there is a need to develop rigorous local screening tools and a more systematic recruitment process.

The diagnostic profile of the study population may not correspond with the patient profile assessed at the clinic as the convenience sampling method assessed a random sample whereby certain diagnostic categories may have been missed. For example, there were lower-than-expected numbers of patients with trauma-related disorders such as PTSD, even though there is a high rate of violence in the community, and therefore, overall higher rates of PTSD might have been expected.

## Conclusion

This study focussed on a vulnerable adolescent population and may be an important vehicle through which to increase both public and clinicians’ awareness of the high prevalence of cyberbullying amongst psychiatrically referred South African adolescents. It provides opportunities for clinicians to review assessment protocols and to incorporate questions and interventions related to supporting adolescents involved with cyberbullying, be they cyber-victims, cyberbullies, or cyberbullies and cyber-victims.

This study focussed on a vulnerable adolescent population referred for the assessment and management of mental disorders. Whilst findings may not be generalisable to the general adolescent population, it may be the vehicle by which to raise clinicians’ awareness of the high prevalence of cyberbullying amongst psychiatrically referred South African adolescents. It may also suggest that assessment protocols may benefit from review and routinely include questions related to cyberbullying, including specific support measures that could be considered to support adolescents involved with cyberbullying. Mental health practitioners assessing adolescents should consider screening for cyberbullying by routinely asking questions relating to both cyberbullying and cyber-victimisation.
